# Humans in the loop: Community science and machine learning synergies for overcoming herbarium digitization bottlenecks

**DOI:** 10.1002/aps3.11560

**Published:** 2024-01-03

**Authors:** Robert Guralnick, Raphael LaFrance, Michael Denslow, Samantha Blickhan, Mark Bouslog, Sean Miller, Jenn Yost, Jason Best, Deborah L. Paul, Elizabeth Ellwood, Edward Gilbert, Julie Allen

**Affiliations:** ^1^ Florida Museum of Natural History University of Florida Gainesville Florida USA; ^2^ The Adler Planetarium Chicago Illinois USA; ^3^ California Polytechnic State University San Luis Obispo California USA; ^4^ Botanical Research Institute of Texas and Fort Worth Botanic Garden Fort Worth Texas USA; ^5^ Prairie Research Institute University of Illinois Urbana‐Champaign Champaign Illinois USA; ^6^ Arizona State University Tempe Arizona USA; ^7^ Department of Biological Sciences Virginia Tech Blacksburg Virginia USA

**Keywords:** citizen science, digitization, humans in the loop, machine learning, natural history collections, Notes from Nature, object classification, object detection, OCR

## Abstract

**Premise:**

Among the slowest steps in the digitization of natural history collections is converting imaged labels into digital text. We present here a working solution to overcome this long‐recognized efficiency bottleneck that leverages synergies between community science efforts and machine learning approaches.

**Methods:**

We present two new semi‐automated services. The first detects and classifies typewritten, handwritten, or mixed labels from herbarium sheets. The second uses a workflow tuned for specimen labels to label text using optical character recognition (OCR). The label finder and classifier was built via humans‐in‐the‐loop processes that utilize the community science Notes from Nature platform to develop training and validation data sets to feed into a machine learning pipeline.

**Results:**

Our results showcase a >93% success rate for finding and classifying main labels. The OCR pipeline optimizes pre‐processing, multiple OCR engines, and post‐processing steps, including an alignment approach borrowed from molecular systematics. This pipeline yields >4‐fold reductions in errors compared to off‐the‐shelf open‐source solutions. The OCR workflow also allows human validation using a custom Notes from Nature tool.

**Discussion:**

Our work showcases a usable set of tools for herbarium digitization including a custom‐built web application that is freely accessible. Further work to better integrate these services into existing toolkits can support broad community use.

The digitization of natural history collections (NHC) has been catalyzed by a combination of best practices in imaging, emerging data standards such as the Darwin Core (Wieczorek et al., 2012), and first‐generation data mobilization tools (e.g., the Integrated Publishing Toolkit; Robertson et al., 2014). Despite this progress, one of the biggest gaps in digitization is converting imaged label information into computable, research‐ready data. This gap, long recognized, has not only persisted but has arguably and surprisingly widened over the past decade as rates of imaging increase but transcription rates remain flat (Vollmar et al., 2010). Outside of imaging, necessary steps in digitization still rely almost entirely on human effort to assemble digital textual records from those images. This has forced institutions to triage, most commonly via production of stub records, where data extracted from labels remain incomplete. For example, the botanically oriented SouthEast Regional Network of Expertise and Collections (SERNEC; https://sernecportal.org/), one of the longest running and expansive Thematic Collections Networks (TCNs), has over 4.9 million specimens imaged and imported into collections digitization aggregators, e.g., Symbiota (https://symbiota.org/) and iDigBio (https://www.idigbio.org/). Of those imaged specimens, 61% still have no locality information as machine‐readable text, greatly reducing the research potential of these data. SERNEC's challenges with digitization completeness are representative of the larger set of issues facing digitization projects (Appendix S1). We also note that all digitization projects listed in Appendix S1 have been active and funded for many years. The ability to speed up digitization activities has an even higher value for new projects where the use of such tools can lead to higher efficiencies by avoiding the need to triage between imaging and downstream digitization.

Overcoming this digitization gap is not insoluble but will require a multifaceted approach that exploits advances in machine learning, while simultaneously leveraging human expertise. Critical here is the concept of a virtuous cycle of improvement via humans in the loop (Xin et al., 2018) through community science efforts. We define the term community science as research where data collection is done by the general public. In brief, human effort is strategically used for building high‐quality training data sets that can be used to automate tasks, and for validating and improving results from initial machine learning models. When leveraging volunteer effort, e.g., via community science activities, a key challenge is making tasks for humans easy and enjoyable enough to accomplish with limited effort but still high value for improving results from automated approaches.

Here, we describe two new services: one for locating typewritten, handwritten, or mixed labels from herbarium sheets and another for extracting text from those labels using optical character recognition (OCR) methods. These services are synergistic and provide a significant advance in semi‐automating digitization of imaged natural history collections. For the label finder and classifier service, we particularly focus on two separate key threads that weave together to lead to superior results. The first is the use of a machine learning–based object detection and classification approach. The second is the importance of Notes from Nature (Hill et al., 2012), a community science project hosted on the Zooniverse platform (https://www.zooniverse.org/), for generating training data for initial models, model validation, and model recalibration. This “human‐in‐the‐loop” framework, especially via an open participatory process, is critical to success but also requires careful attention to data filtering and user experience design.

For the OCR service, we focus on the importance of fine‐tuning OCR results via a workflow that uses multiple parallel steps ordered so as to reduce error. While OCR is used in museum digitization (Tulig et al., 2012; Drinkwater et al., 2014), the quality has been highly variable, and most natural history museums do not have the resources, time, or training to train their own models. Although existing open‐source OCR engines can have remarkable results in controlled settings, their default settings do not always handle text detection in the myriad of conditions, fonts, and stray markings often found on NHC labels (Heidorn and Wei, 2008). While commercial OCR services may help to remove some of these issues, they are often too costly for museum budgets. Additionally, image preprocessing steps to enhance the OCR results are not uniformly appropriate for all sheet conditions. Each sheet may require a different image processing technique to prepare it for OCR, based on factors such as the clarity of the text on the label or the slant of the label. To reduce this inherent noisiness, we used an ensemble pipeline (see below) that focuses on OCR pre‐processing and post‐processing steps. As with object detection, humans‐in‐the‐loop processes also provide a key means to help improve outputs via a newly developed OCR correction tool deployed on Notes from Nature.

One of the challenges with the deployment of semi‐automated approaches more generally is evaluating how much time and effort are saved using them compared to manual effort, and how much improvement in quality is possible (Groom et al., 2019). Key to our efforts has been quantifying rates of success and fine‐tuning our results based on those quantifications. We also rigorously evaluated the steps that produce the best possible OCR outputs, and this rigor provides a means to quantify how much better our ensemble models are compared to simpler solutions. We provide a working demonstration of the services via an interactive web application for further exploration. In sum, below we provide key details on two new services that can already be integrated into established community tools such as Symbiota to markedly improve the rate and quality of digitization of natural history collections, and discuss next steps for parsing records and integrating results broadly into existing internet‐scale tools for managing NHC data.

## METHODS AND RESULTS

### Object detection and classification

#### Generating training data

We collected training data through volunteer effort in the Notes from Nature project for object detection and classification. Notes from Nature is hosted on the Zooniverse platform and engages volunteer participants around the world by bundling images and metadata into “expeditions.” Expeditions are composed of a discrete set of images, one or more tasks that participants are asked to complete, and a set of instructions to direct users as to how the tasks should be completed. Participants then perform the tasks on the related images to generate the training data set. In most Notes from Nature expeditions, we have focused on transcription tasks, but for training data development we set up a very different set of classification tasks for users.

**Figure 1:**
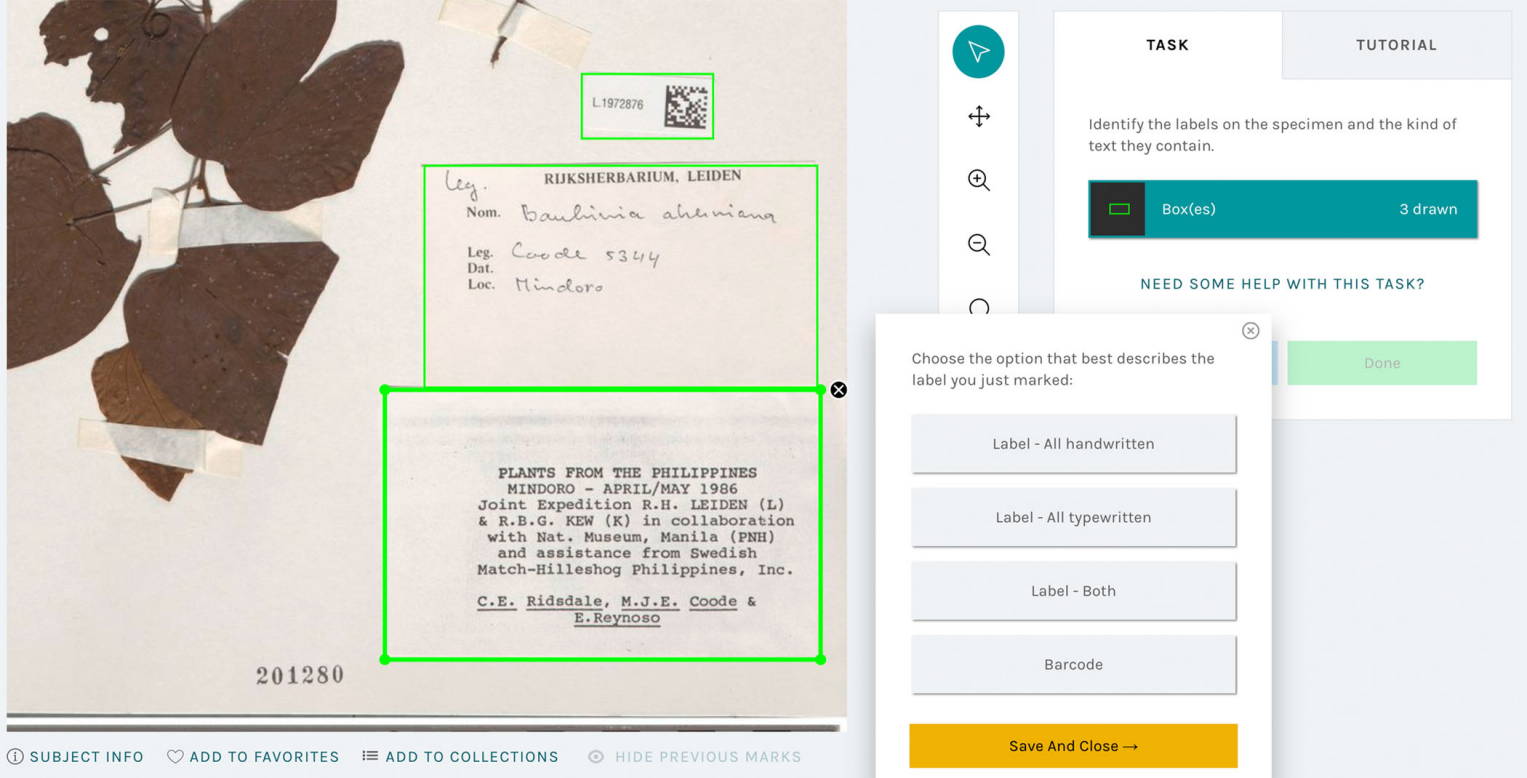
The Notes from Nature user interface. The green boxes show where the three different labels were identified by a participant. The dialog box allows the user to specify the type of label.

We ran two different Notes from Nature expeditions related to gathering training data for building object detection and classification models. The first expedition asked participants to draw boxes around all the labels they saw on a herbarium sheet and indicate the type of information contained on the labels: handwritten, typewritten, or both (Figure 1). We defined a label as anything that was added to the sheet that was clearly neither the sheet nor the plant, but excluding stamps. We also asked participants to classify the label as a main label or a barcode, which is often found on herbarium sheets.

**Figure 2:**
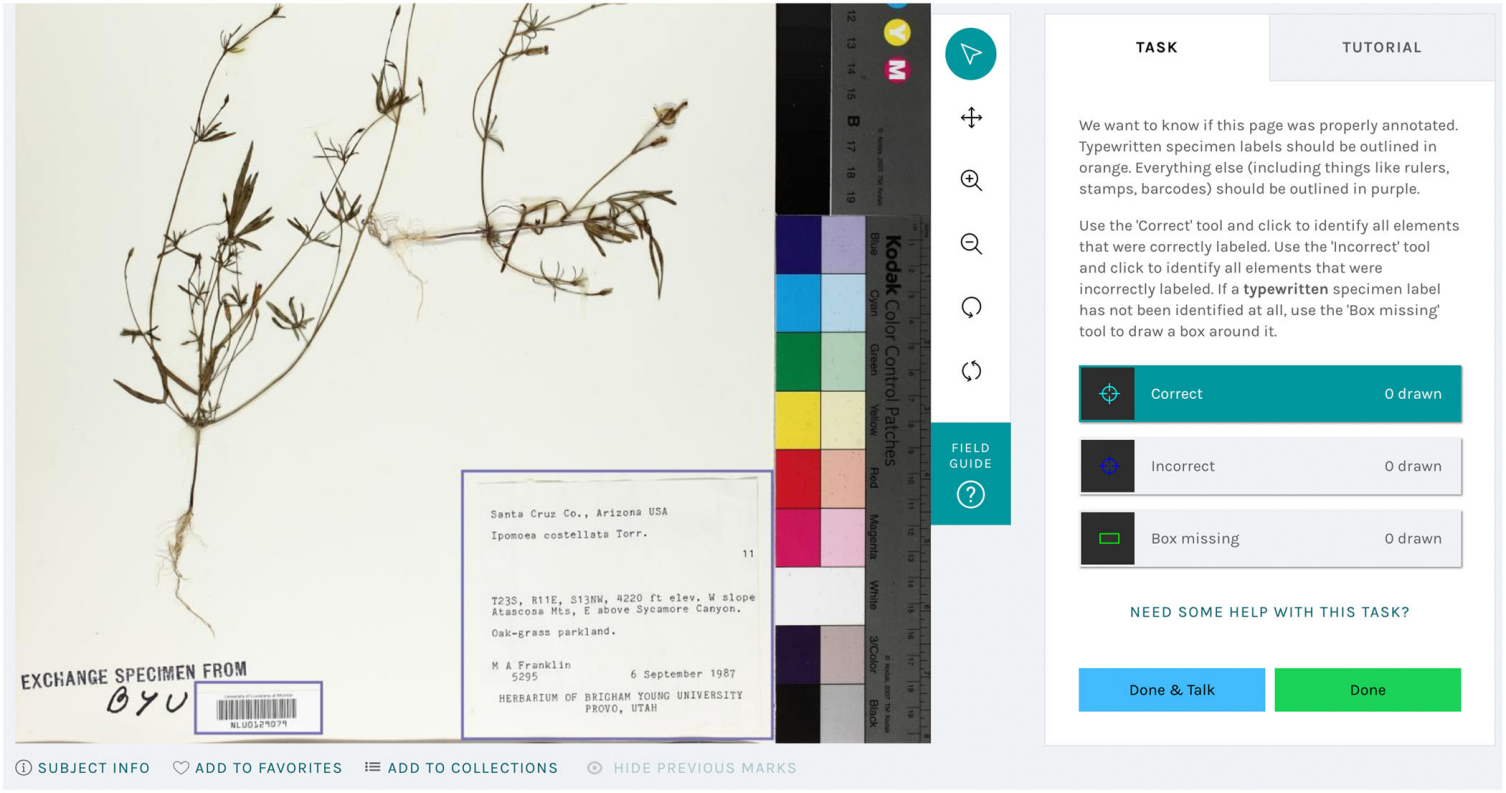
The Notes from Nature user interface for Label Babel 3 ‐ Rise of the Machines. Typewritten labels should be outlined in orange and all other labels, such as barcodes, in purple. For this specimen, the user will use the “Incorrect” tool to correct the label in the lower right of the specimen image.

We gathered two random sets of images of herbarium sheets from SERNEC and used these images as training data. The first contained 4995 images and the second contained 3000. SERNEC represents one of the oldest and largest repositories for herbarium specimens; with currently 125 herbarium collections, it likely represents much of the diversity of herbaria sheets in English. Notes from Nature expeditions have a set “retirement limit” that defines how many unique participants must complete the requisite task(s) on an image before it is considered complete and is retired from the set. We set the retirement limit to three for this expedition to allow for majority decision‐making. Expeditions are named and advertised on the main Notes from Nature landing page. Because the training data development process postceded a first attempt at finding labels called “Label Babel,” we named the expeditions “Label Babel 2” and “Label Babel 3.” Once launched, we promoted the expedition broadly via a blog about the effort (Denslow, 2022) hosted on the Notes from Nature platform, and the expedition was also featured in a newsletter to the Zooniverse community. Label Babel 2 ran for 80 days and had 416 participants. Figure 1 shows a view of the expedition tasks, and Figure 2 provides a view of the types of outputs that were generated for an exemplar sheet. Once the expedition was complete, we used a set of Python scripts to generate a reconciled consensus set of the boxes and classifications of box types (typewritten, handwritten, both, barcode, or other). These data served as input into the machine learning–based computer vision workflow discussed below.

#### Improving detection and classification via humans‐in‐the‐loop corrections

The initial run of our object detection and classification machine learning pipeline, while credible, had room for significant improvement; success rates for detection and classification of labels were around 75%, which is too low for broad use. In order to improve model results, we developed a new Notes from Nature expedition that utilized the test and validation data sets and had a different set of task requirements. This new expedition, which we cheekily named “Label Babel 3 ‐ Rise of the Machines,” shows participants an imaged herbarium sheet with machine learning–based predictions of where labels are located and their type, drawn as boxes colored per type (Figure 2). Participants are then asked to annotate, per sheet, which labels were correctly and incorrectly labeled, or if a typewritten label was completely missed. The annotation process involved using a “correct” or “incorrect” tool, which allows users to click inside a label and add that annotation.

Label Babel 3 ran from 2 June 2022 through 24 July 2022 (56 days) and involved 177 participants. Once the expedition was complete, we utilized a custom script to check and clean the results before downstream use. In particular, this script checked for cases where participants annotated outside the known position of the labels (a small percentage), and these were removed. The quality‐controlled final set of corrected labels was then fed back into the object detection, segmentation, and classifier model and tested with a new set of herbarium sheets to determine performance, as described below.

#### Developing an object detection and classification pipeline

We developed initial machine learning models using training data generated from Label Babel 2. The first step in this process was cleaning and collating the training data. For each imaged herbarium sheet, we assembled an “average” outline for each label on each imaged herbarium sheet from the three independent outlines generated by participants. In cases where one outline was very different from the others, we removed that as an outlier and averaged the other two. We also scored the majority rule classification of the label type (e.g., handwritten, typewritten, both, or barcode). We then used the final validated input data for testing two different transfer learning frameworks: efficientdetv2_d0 (Tan and Le, 2021) and Faster R‐CNN (resnet50 FPN; Ren et al., 2017). We tested the quality of these models using the intersection over union (IoU) metric. This takes the outline of a label that has been correctly identified (by type) and compares how much it overlaps with a ground truth label (e.g., one in the testing or validation set) by measuring the area of overlap over the total area of the two labels.

While initial model runs were relatively performant, we were unsatisfied with the overall performance and opted to continue improving these models. In order to do so, we used inputs from the Label Babel 3 expedition, which gathered human‐validated corrections, to assemble an improved data set for further model training. We tested previous models used in our first attempt at object detection and classification, as well as a You Only Look Once (YOLO) model (YOLOv7; Wang et al., 2022), and opted to use the YOLOv7 model to both find and identify labels on a herbarium sheet, given its speed and performance (Table 1). This model was trained on a laptop with an 8‐GB Nvidia GPU (GeForce RTX 2080 Mobile; Nvidia, Santa Clara, California, USA). We used a 60%/20%/20% split for training, validation, and testing data sets, and used validation‐based early stopping (Prechelt, 2012) for determining how long to train the model, which resulted in 100 epochs of training.

**Table 1:** Machine learning model parameters and performance, including transfer models used and input training data sets, and size of image. We tested how well our calibrated model performed via comparison with the test and validation data sets, using average intersection over union (IoU) for object detection as a success metric. We were able to improve model performance using corrected data and a YOLOv7 transfer model, which is also fast to calibrate.

Model	Training data set	Epochs	Image size (pixels)	Average IoU	Notes
YOLOv7	Label Babel 3	100	640 × 640	0.8228	Best model
YOLOv7	Label Babel 3	50	640 × 640	0.8135	
YOLOv7x	Label Babel 3	100	640 × 640	0.7939	
YOLOv7x	Label Babel 3	100	800 × 800	0.7911	
efficientdetv2_d0	Label Babel 2	200	512 × 512	0.7720	
Faster R‐CNN (resnet50 fpn)	Label Babel 2	100	224 × 224	0.6167	Retrained with newer data
Faster R‐CNN (resnet50 fpn)	Label Babel 1	40	224 × 224	0.3602	2021 pilot

After producing the final model, two co‐authors (R.G. and J.A.) validated the model results on a randomly selected set of 923 imaged herbarium sheets from which labels were detected and classified. We tested whether main labels were correctly detected and classified, and found that the model had correctly performed this dual task on 93.9% of the images. The most common reason for failure was misclassification; for example, of the 6% of labels that were misclassified, those with significant proportions of handwriting were classified as being entirely handwritten. There were also cases (~5% total) where the model located and classified the main label correctly, but had other issues such as “phantom labels” (i.e., predicted areas annotated by the model as a label that were not). In a small subset of cases (20 in total), we skipped assessing the model performance because of issues with the input image (very low quality or otherwise not usable for testing performance).

### An improved OCR pipeline for specimen labels

#### Developing an OCR pipeline for converting to digital text

The main goal of the OCR pipeline is to improve OCR of imaged, typewritten labels that are extracted in the object detection and classification steps described above. The OCR pipeline starts by performing any of four image processing techniques that often help the OCR process: (1) Apply OCR to an unmodified label image; this sometimes works with newer herbarium sheets. (2) Slightly blur the image, scale it to a size that works with many OCR images, orient the image so it is right side up, and then deskew the image to fine‐tune its orientation. (3) Perform all of the steps in #2 and additionally perform a Sauvola binarization (Sauvola and Pietikäinen, 2000) of the image, which often helps improve OCR results. (4) Perform all of the steps in #3, then remove “snow” (image speckles) and fill in any small “holes” in the binarized image.

These pre‐processing steps generate up to four separate images of each label that are used as input. Each of these images is then run through either Tesseract version 4.1.1 (Smith, 2007) and/or EasyOCR version 1.6.2 (https://github.com/JaidedAI/EasyOCR), an open‐source OCR engine, yielding one to eight OCR text outputs. These texts will vary, sometimes significantly. Before we find the consensus sequences, we remove any outlier texts (i.e., those with a Levenshtein distance greater than a predetermined cutoff using a threshold of 128 determined via trial and error) from the two closest sequences. This prunes complete failures resulting from the OCR process. We also correct some errors commonly associated with the OCR engines, such as adding spaces before punctuation like periods or commas, or common character substitutions.

The next step in the workflow is to use a multiple sequence alignment that is directly analogous to the ones used for biological sequences (Gusfield, 1997), but instead of using a point accepted mutation (PAM) matrix or blocks substitution matrix (BLOSUM), we use a visual similarity matrix. Visual similarity is determined based on the font, thus an exact distance is not feasible. Instead, we use a rough similarity score that ranges from +2 (for characters that are identical) to −2 (for characters that are different in shape, e.g., a period vs. a W), a gap penalty of −3, and a gap extension penalty of −0.5. Visual similarity values were based on exploratory analyses to determine clear thresholds, and gap and extension penalties were heuristically determined. After the consensus sequence is obtained, a final text cleanup step is performed to fix spelling and common OCR errors (e.g., the addition or removal of spaces within words, or common character substitutions).

**Figure 3:**
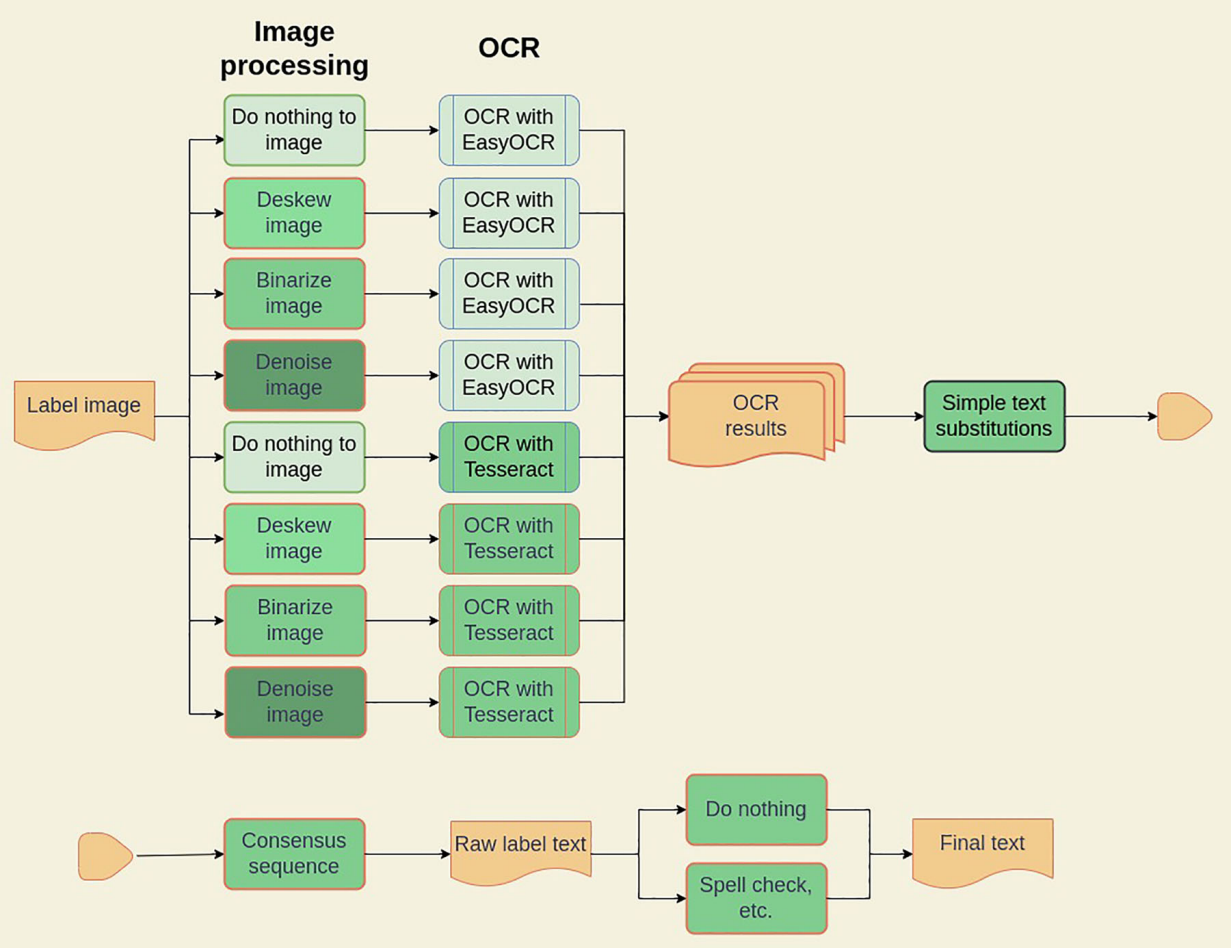
Processing steps used in the OCR pipeline. Label images are fed into image processing pipelines and then to two separate OCR engines. The OCR results are checked for simple text substitutions and then fed into an algorithm to generate a consensus sequence, which generates raw label text. Post‐processing steps are applied to generate the final text.

The OCR pipeline has multiple parameters to tune, not the least of which is what image processing techniques work best, on average, with each OCR engine. We therefore generated a large set of OCR pipeline permutations (510 total) and generated OCR results for each of these permutations. Figure 3 shows all the processing steps and how they were chained together. We tested which permutation worked best via comparison with a gold standard, discussed below.

#### Testing the OCR pipeline via an independent gold standard

We generated a volunteer‐produced “gold standard” data set to determine the best OCR workflow. We first developed a clear rubric explaining exactly how to reproduce content on labels verbatim. To generate the highest quality transcriptions, we then recruited two volunteers to score labels independently and crosscheck each other's work. Because we wanted to be sure that transcriptions from the gold standard could be used to test the quality of OCR workflows, the rubric for transcribing labels made especially clear that labels must be scored verbatim (i.e., errors on the label should not be corrected). The two volunteers scored a total of 337 imaged labels to serve as the gold standard. After the gold standard data set was complete, we determined the error rate for each permutation of our OCR pipeline; the pipeline with the fewest cumulative errors over the entire gold standard was determined to be the most successful. Table 2 provides a summary of the performance of a subset of the pipeline permutations.

**Table 2:** A subset of OCR workflow permutations used. The best OCR workflow always included pre‐ and post‐processing steps, and while there was a best permutation that included multiple steps, many workflows were in range of the best one.

Total OCR errors^a^	EasyOCR	Tesseract	Deskew then EasyOCR	Deskew then Tesseract	Binarize then EasyOCR	Binarize then Tesseract	Denoise then EasyOCR	Denoise then Tesseract	Post process
1881	Y	Y	Y	Y	N	Y	N	Y	Y
1892	N	Y	Y	Y	Y	Y	N	Y	Y
1903	Y	Y	Y	Y	Y	Y	N	Y	Y
3146	Y	Y	Y	Y	Y	Y	Y	Y	N
6632	Y	Y	N	N	N	N	N	N	Y
7230	Y	Y	N	N	N	N	N	N	N
7455	N	Y	N	N	N	N	N	N	N

^a^ Total OCR error is ranked from lowest to most errors for these permutations and was determined via assessment of 337 gold standard labels.

#### OCR correction via public participation

**Figure 4:**
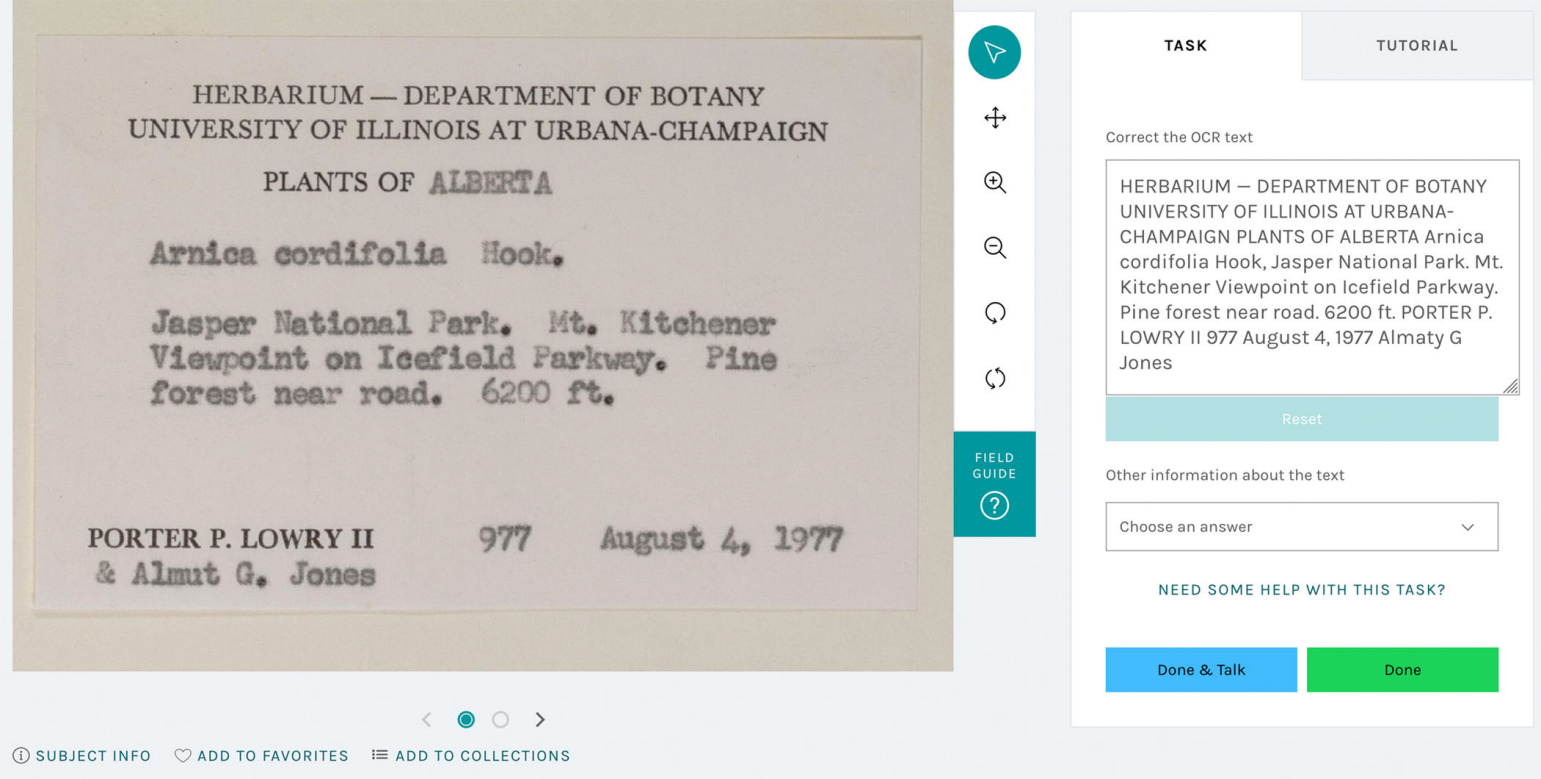
The Notes from Nature user interface for the OCR correction expedition. The original label is on the left, and a prepopulated and editable text box is on the right. The user can correct the text, as well as provide other information about the text before completing the task and moving to the next label.

Despite the improved OCR results obtained using our workflow, OCR still requires correction in some cases, and these corrections can ultimately inform further improvements in automated approaches. We again use a community science humans‐in‐the‐loop approach, via development of a bespoke OCR correction tool on Notes from Nature. This new tool provides volunteers with images of labels next to a prepopulated text box containing the OCR output. Participants are asked to directly edit the text to make any corrections of that OCR output. Participants are also given the option to provide additional information about the label (e.g., if it is a barcode, stamp, or ruler). We prototyped this tool in a beta launch and used feedback from volunteers to improve some usability aspects, before launching the tool for general use in autumn 2022 (Figure 4).

### Services and web application for object detection, classification, and OCR

**Figure 5:**
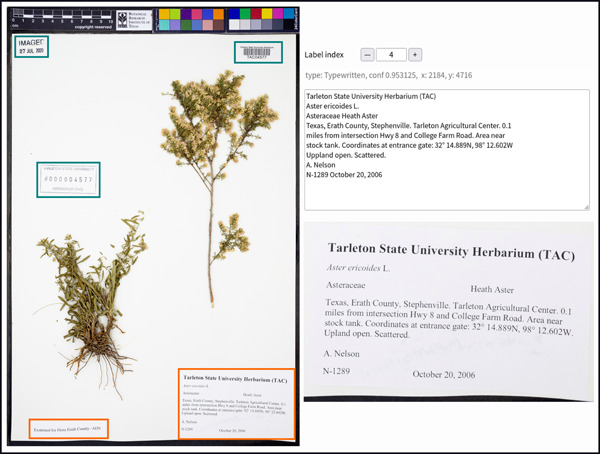
An example of data returned from an imported digitized herbarium sheet. The application provides detected labels and their extents along with a classification of label types. The user can select which labels are then fed to the OCR pipeline, which returns an OCR result for the label of interest.

We created a Python FastAPI web application usable for testing the label finder and OCR ensemble approach. We created both server endpoints and a client web page that can be used to access the server; these services are best described via the client interface. First, a user selects an image of a herbarium sheet on their drive; alternatively, they can select a random sample sheet from a set of images available on the site. The user then sends the sheet image to the server (/find‐labels endpoint), which uses the trained neural network to find the labels and return their type and coordinates in JSON format. The client presents them as colored boxes drawn on the sheet image (Figure 5). Typewritten labels eligible for OCR appear as orange boxes, while any labels that cannot be identified using OCR appear as teal boxes. The client allows the user to correct any labels they think were misidentified by the neural network. When the user is satisfied with the labels, they can send the sheet and the JSON coordinates to the /ocr‐labels endpoint; the server will then use the OCR ensemble on every typewritten (orange) label on the sheet and return a new JSON object with the resulting text (see Figure 5). The user can edit the text if there are problems with the OCR. The client allows all label images to be saved, along with the JSON object with the text from the OCR. The web app is available for testing at: http://3.89.120.132/ and is running on the Amazon Cloud.

## DISCUSSION

An overarching obstacle for developing automation tools for the digitization of natural history collections is the vast heterogeneity in how specimens and their associated analog data and metadata have been stored over hundreds of years of collecting and curating (Hedrick et al., 2020). That span of time is long enough to cover major revolutions in how printed content is produced, from handwritten labels to typewriters to laser printing. Even focusing just on herbarium sheets, where there are shared practices in how labels are affixed to sheets and what information is reported on those labels, there are still significant challenges (Tulig et al., 2012). These include label position and formatting, the different types of devices used to print the labels, different fonts (including historic ones that are no longer used), and a myriad of other issues ranging from how the paper and print on labels age to organismal damage to labels resulting from fungi and bacteria. Because of these issues, there is no single set of automated approaches for digitization that works best for all input sheets. Furthermore, we are aware of no single automation solution that can be guaranteed to produce consistently low error rates across all labels. Because of these challenges, OCR error rates for herbarium sheet labels are often high, just as Tulig et al. (2012) noted a decade ago. At best, OCR is sometimes used to capture a first snapshot of a label that can be stored and searched (Tulig et al., 2012), but this likely provides only a marginal time savings to collections staff.

We argue that the work presented here shifts the needle toward semi‐automation providing a significant savings in time and effort, while recognizing the critical value of humans in the loop. For the foreseeable future, some labels are likely to not be amenable to automated approaches at all, and most labels will require some level of human effort to finalize. However, the services we unveil here are major steps forward for reducing the cost and time to convert typewritten labels, which can be sorted from handwritten and mixed labels performantly and with high accuracy. We know that these tools are significant improvements over current practices because we have carefully assembled gold standard data sets that provide a means to both determine performance and directly compare these tools to more generic options. We advocate for rigorous external validation of performance and error rates; this is especially critical for ongoing improvements of tools.

Our fully open‐source workflow for converting labels to text revolves around finding the main label on a herbarium sheet and passing it to a OCR pipeline optimized for the vagaries of NHC data. For label detection and classification, we tried several different neural network architectures; however, a simple YOLOv7 model (Wang et al., 2022) pre‐trained on a Common Objects in Context (COCO) data set (Lin et al., 2014) performed well (IoU = 82%, correct detection of typewritten main labels >93%) after only 100 epochs of training. This is likely because the model is only required to find squares with text on a herbarium sheet and OCR that text. The model is by no means perfect, and one area where improvement is still needed is adjusting the label finder's confidence parameters to remove ghost labels or add missing labels. While we were able to dramatically improve results via a humans‐in‐the‐loop correction process, more training data are likely to further increase the quality of results. Nevertheless, our validation efforts show that the accuracy is high enough for general use, as the label information (date, locality, species) was accurate.

Our OCR efforts showcased the development of a bespoke pipeline for labels that reduces error rates significantly, by a factor of approximately four, from simply using off‐the‐shelf open‐source OCR. We first tried to find a single best image‐processing algorithm coupled with a single best OCR engine, but the results were unsatisfactory. Some image processing techniques work well for some labels but not for others, and the two open‐source OCR engines we leveraged here have unique strengths and weaknesses. In effect, we are performing as many combinations of image processing and OCR engines as practical and combining the OCR texts into a consensus text using multiple sequence alignment. To further lower error rates, it will be useful to try different image processing techniques and newer OCR engines (e.g., GOCR and Kraken). It may also be possible to improve how the consensus sequence is generated. Currently, texts that are significantly different from the rest of the ensemble are discarded, but all remaining texts are given equal weight. It may be possible, however, to weight texts (or parts of the text) to improve OCR scores against the gold standard. Another area of possible improvement is to improve the OCR text post‐processing. The spell checker is currently geared toward herbarium specimens, but the list of common substitutions is not extensive or weighted in any way; improving this could help further reduce errors. Finally, our OCR correction process is currently set up to provide a simple way to curate OCR results and assure that errors are corrected as expeditiously as possible. Those OCR corrections could potentially also be fed back into the pipeline as a means of improving results, either via directly improving the model underlying OCR engines or as a downstream step where common OCR errors can be detected and fixed (Barber et al., 2013).

Critical to the success of this pipeline has been the recognition that community science tools can be deployed at multiple stages across the overall workflow (e.g., humans in the loop; Chen et al., 2023) to help either scale up production of training data (critical for the label finder effort) or for data correction and improvement (both for finding and classifying labels and for OCR). This approach best uses human effort but requires thought and care in assuring that tasks are simple enough while still being engaging, and that documentation is as clear as possible to cover any corner cases or other challenges. In designing Notes from Nature expeditions, we often tested assumptions about usability via launching closed “alpha‐test” expeditions, in which a select few volunteers participated and provided feedback, before launching the expedition more broadly. We also note the very strong need for quality control to improve the outputs of such efforts. When we initially screened results from our Label Babel 3 expedition focused on correcting label detection and classification, we noted spurious records that were greatly reducing performance. When these rare problem cases were removed, our results dramatically improved.

We close by noting next‐step activities in this area that we feel are nascent but where quick progress is likely in the next few years. First, our current workflow removes handwritten labels from our pipeline, but it is possible that in the next few years handwriting recognition will improve to the point where it may be feasible to process such labels. The challenge again is heterogeneity in handwriting legibility and quality, which may prove more intractable. Second, we see quick advances in parsing labels into Darwin Core fields (Wieczorek et al., 2012). Some prototypes have been attempted (Owen et al., 2020), but scalable and cost‐effective approaches to this may come from outside our field as OpenAI tools such as ChatGPT have already shown to be able to successfully create parsed Darwin Core content through our explorations. Even if artificial intelligence provides a performant solution for parsing, we see a real need for community science tools for correcting parsing outputs generated via automation. These correction tools assure a human curation step and help improve the ability of machine learning models to have high‐quality data for training better models. Carefully designed tools on Notes from Nature can decrease the time and effort for parsing, providing both scalability and lowering effort costs. Finally, we note the pressing need to integrate automation services more fully into tools already in the broadest use in the community. These services may most profitably fold into existing content and tool management systems such as Symbiota (Gries et al., 2014). Such integration can also help assure standardization and metadata reporting about how outputs were generated, which is critical but still lacking (Schelter et al., 2017). For example, each corrected label obtained via OCR should include a report about the steps used to create it, including the versions of the tools used. With the work described here and strategic efforts moving forward, we foresee continuing advances in leaping the gaps that have hampered digitization over the past decade, not only for herbarium specimens but for other groups with enormous digitization backlogs (e.g., insects).

## AUTHOR CONTRIBUTIONS

R.G., M.D., R.L., and J.A. designed the study, with support from S.B. R.L. developed the code and machine learning tools while M.D. was instrumental in setting up Notes from Nature (NFN) expeditions. S.B. led the coordination of NFN tool development with M.B. and S.M. R.G. and J.A. wrote the first draft with support from R.L. and M.D. J.Y., J.B., D.L.P., E.E., and E.G. helped with writing and editing, and all authors approved the final version of the manuscript.

## Supporting information


**Appendix S1**. State of digitization of specimen labels across major botanical collections found in North America.

## Data Availability

All code is open source and available at https://zenodo.org/record/7502402 (LaFrance, 2023a) and https://github.com/rafelafrance/digi_leap/tree/v0.1.1, which includes all specific scripts mentioned in the text. Data used in creating models are available at https://zenodo.org/records/8111407 (LaFrance, 2023b) and https://github.com/rafelafrance/digi_leap/tree/v0.1.2.
