# Supplementary material for: Humans in the loop: Community science and machine learning synergies for overcoming herbarium digitization bottlenecks
Source: Appl Plant Sci. 2024 Jan 3;12(1):e11560. doi: 10.1002/aps3.11560 (PMC10873811; doi:10.1002/aps3.11560)
Supplement: Supplementary file 1 — Appendix S1. State of digitization of specimen labels across major botanical collections found in North America. [file APS3-12-e11560-s001.docx]

**Appendix S1.** State of digitization of specimen labels across major botanical collections found in North America.

SEINet is a portal that serves records from many collections from around the United States, Canada, and Mexico. Of the records available through SEINet, most of which were digitized on grants that are now completed, nearly 20% are missing collector and locality data, and nearly 60% lack mappable coordinates. The Botanical Research Institute of Texas (BRIT) has acquired collections from the Vanderbilt University herbarium (VDB) and the herbarium at the University of Louisiana at Monroe (NLU), of which 90% are missing locality and coordinates.

| **SEINet Regional Consortium** | **No. of collections** | **No. of specimens** | **Missing**  **collector** | **Missing full locality** | **Missing coordinates** |
| --- | --- | --- | --- | --- | --- |
| Arizona - New Mexico Chapter | 34 | 1,231,911 | 34,254 | 31,580 | 324,129 |
| Canadian Herbaria | 3 | 265,329 | 25,552 | 69,865 | 140,585 |
| Consortium of Midwest Herbaria | 55 | 2,723,707 | 205,356 | 550,367 | 1,810,759 |
| Consortium of Northeastern Herbaria | 4 | 1,390,384 | 204,494 | 281,439 | 1,278,923 |
| Intermountain Regional Herbaria Network | 27 | 863,063 | 129,914 | 139,107 | 529,771 |
| Mid-Atlantic Herbaria | 17 | 2,623,876 | 104,889 | 280,337 | 1,300,886 |
| North American Network of Small Herbaria (NANSH) | 13 | 195,977 | 73,581 | 127,060 | 195,610 |
| Northern Great Plains Herbaria | 23 | 1,099,650 | 39,839 | 85,708 | 564,876 |
| Pacific Herbaria | 18 | 2,110,138 | 84,251 | 89,167 | 617,500 |
| Red de Herbarios de México | 27 | 172,186 | 405 | 4,264 | 32,463 |
| Southern Rocky Mountain Herbaria | 24 | 1,464,252 | 75,687 | 153,576 | 459,460 |
| Texas Oklahoma Regional Consortium of Herbaria (TORCH) | 28 | 2,458,677 | 837,448 | 971,482 | 2,074,551 |
| **Totals** | **273** | **16,599,150** | **1,815,670** | **2,783,952** | **9,329,513** |
